# 抗体类新药治疗复发/难治性弥漫大B细胞淋巴瘤的研究进展

**DOI:** 10.3760/cma.j.issn.0253-2727.2023.03.016

**Published:** 2023-03

**Authors:** 彭鹏 许, 维莅 赵

**Affiliations:** 上海血液学研究所，医学基因组学国家重点实验室，国家转化医学中心（上海），上海交通大学医学院附属瑞金医院血液科，上海 200025 Shanghai Institute of Hematology, State Key Laboratory of Medical Genomics, National Center for Translational Medicine（Shanghai）, Hematology Department of Ruijin Hospital Affiliated to Shanghai Jiaotong University School of Medicine, Shanghai 200025, China

2019年我国新增霍奇金淋巴瘤（HL）9 468例，新增非霍奇金淋巴瘤（NHL）91 954例。HL的发病率和死亡率分别为0.57/10万和0.15/10万，而NHL的发病率和死亡率更是达到4.99/10万和2.32/10万[1]。弥漫大B细胞淋巴瘤（DLBCL）是最常见的淋巴瘤亚型，占所有淋巴瘤的35.8％[2]。随着规范化治疗的推广和新药的不断研发，DLBCL患者的生存获得了很大的改善。中国DLBCL患者的5年总生存（OS）率达到38.4％[3]，其中一线接受利妥昔单抗联合含蒽环类药物化疗方案患者5年、10年OS率分别为69％、55.6％，接近欧美国家水平[4]。约60％的DLBCL初治患者可通过R-CHOP免疫化疗方案治愈。然而复发/难治性DLBCL（R/R DLBCL）患者接受现有的挽救化疗方案联合自体造血干细胞移植（ASCT）巩固治疗仅有10％的治愈率[5]，不适合行ASCT或ASCT后复发患者的预后更差，中位生存期仅6～12个月左右[6]–[7]。近年来，虽然嵌合抗原受体T（CAR-T）细胞治疗的出现使部分R/R DLBCL患者获得持久缓解与长期生存，但是CAR-T细胞治疗高昂的费用、较长的制备周期、特殊的不良反应等诸多因素限制了其在临床的应用。抗体药物偶联物（ADC）、单克隆抗体和双特异性抗体等抗体类新药的研发与应用为R/R DLBCL的治疗打开了新局面。本文对这些抗体类新药或治疗方案在R/R DLBCL治疗领域的临床研究进展进行归纳和总结。

一、抗体药物偶联物（antibody-drug conjugate, ADC）

ADC是一类通过特异性的单克隆抗体与杀伤性的细胞毒性药物偶联起来的靶向生物制剂，以单抗为载体将小分子细胞毒性药物高效地运输至目标肿瘤细胞。ADC药物结合了靶向性、选择性强的抗体和抗肿瘤活性细胞毒性药物的优势，在保留小分子细胞毒性药物肿瘤杀伤特性的同时，降低小分子细胞毒性药物的脱靶不良反应，提高了抗肿瘤治疗的获益风险比。目前，DLBCL领域已有两款ADC药物获批治疗R/R DLBCL，分别是Loncastuximab tesirine（Lonca）和Polatuzumab vedotin（Pola），同时还有多个产品的研究正在进行。

1. Lonca：Lonca是由人源化抗CD19单克隆抗体通过蛋白酶可裂解的连接子与吡咯并苯并二氮杂卓（PBD）二聚体细胞毒素偶联而成。Lonca与表达CD19的DLBCL细胞结合后被细胞内吞，并释放细胞毒素-PBD二聚体，该毒素不可逆地与DNA结合抑制DNA复制导致肿瘤细胞死亡。与常见的微管抑制剂毒素相比，PBD二聚体具有更优秀的细胞杀伤作用。

Lonca单药治疗复发难治B细胞NHL的疗效和安全性已被证实[8]。研究纳入183例复发难治B细胞NHL患者，包括139例R/R DLBCL患者，中位年龄63岁，其中“双/三打击”占16.6％，中位治疗线数为3，一线原发耐药患者占21.6％。在经过多重治疗的DLBCL患者中，Lonca单药治疗客观缓解率（ORR）达42.3％，完全缓解（CR）率为23.4％，中位无进展生存（PFS）时间为2.8个月，中位OS时间为7.5个月。最常见的不良反应是血液学不良反应、疲乏、恶心、水肿以及谷氨酰转肽酶升高。在另一项Ⅱ期单臂试验LOTIS 2中，145例R/R DLBCL成人患者既往至少接受过2次系统性治疗，中位年龄66岁，既往治疗的中位线数为3，63％为难治性，17％为ASCT失败患者，9％接受过CAR-T细胞治疗，Lonca单药治疗的ORR为48.3％，CR率为24.1％，部分缓解（PR）率为24.1％。缓解患者的中位缓解持续时间为13.4个月[9]。在高级别B细胞淋巴瘤（HGBCL）、组织学转化的淋巴瘤及ABC亚组中均具有类似的反应率，ORR分别为45.5％、44.8％和47.8％。另外，在之前接受过CAR-T细胞治疗的患者中，Lonca的ORR为46.2％。最常见的3级以上的不良反应为血液系统相关反应。因此，2021年美国FDA首次批准了Lonca单药用于治疗已接受2种或多种系统疗法的复发或难治性大B细胞淋巴瘤成人患者。Lonca也成为全球首个、也是迄今唯一一款获批上市的CD19-ADC药物。

LOTIS-3研究探索Lonca联合伊布替尼治疗R/R DLBCL的疗效。目前联合用药的ORR达到57.1％，CR率为34.3％。GCB亚型患者疗效更加显著，ORR达76.9％，CR率为46.2％，Lonca与伊布替尼联合具有可控的不良反应[10]。

2022年ASCO大会上公布了一项正在进行的国际多中心Ⅲ期临床试验——LOTIS-5研究[11]，旨在比较R/R DLBCL患者应用Lonca与利妥昔单抗联合方案和利妥昔单抗联合吉西他滨、奥沙利铂（R-GemOx）免疫化疗方案的疗效与安全性。LOTIS-5研究是全球首个在R/R DLBCL二线治疗中利用ADC药物完全替代化疗方案的Ⅲ期注册性临床试验，该研究的患者招募正在进行中，中国地区的多个研究中心也正在陆续启动中。

2. Pola：Pola是一种抗CD79B单克隆抗体，通过蛋白酶可裂解的连接子与微管抑制剂单甲基奥瑞他汀E（MMAE）偶联。CD79B作为B细胞受体复合物的成分，是B细胞NHL的治疗靶标。

Pola单药治疗复发难治B细胞NHL和慢性淋巴细胞白血病的疗效与安全性已经被一项全球多中心Ⅰ期研究（NCT01290549）证实[12]。在建议Ⅱ期剂量2.4 mg/kg下，ORR为56％。中位无复发生存时间为5个月，中位缓解持续时间为5.2个月。最常见的不良反应是中性粒细胞减少、贫血和周围感觉神经病变[12]。

Pola联合利妥昔单抗和苯达莫司汀（Pola+BR）的疗效也在不适合行ASCT的R/R DLBCL患者（GO29365）中被认可。Pola+BR治疗组的ORR（45％对17.5％）和CR率（40％对17.5％）高于BR单药治疗组[13]，且PFS和OS都得到了显著改善。仅10％的患者发生3～4级不良事件（AE），主要是感染和血细胞减少[14]。基于该结果，2019年美国FDA批准了Pola与BR的联合方案用于既往至少接受两次治疗的R/R DLBCL患者。

研究者为了探讨Pola与其他治疗方案联用的可行性，开展了一项Ⅲ期、多中心、开放标签、随机临床试验——POLARGO研究[15]，旨在比较Pola-R-GemOx和R-GemOx治疗R/R DLBCL患者的安全性和有效性。2022年ASCO和EHA大会上报道了POLARGO研究安全性导入阶段的初步分析结果。安全导入阶段共入组15例患者，其中11例（73％）患者接受≥4个周期Pola-R-GemOx治疗。入组患者中位年龄为76（47～87）岁，10例（67％）患者IPI评分为3～5分，7例（47％）患者既往接受≥2线治疗，8例（53％）患者最后一次治疗难治。15例患者中只有5例患者完成8个周期的治疗。常见不良反应包括外周神经病变（53％）、便秘（33％）和疲劳（33％）。33％的患者发生3～4级AE，以血液学不良反应为主。未发生5级AE。15例患者在治疗结束时的ORR为40％，CR率为27％。

3. 维布妥昔单抗（Brentuximab vedotin, BV）：BV是一种靶向CD30的ADC药物，由CD30单克隆抗体共价连接抗微管药物即MMAE组成。BV已在经典型HL及部分CD30阳性T细胞淋巴瘤中获批多项适应证。在DLBCL领域也开展了一系列研究。

BV单药或BV联合利妥昔单抗治疗复发难治CD30阳性DLBCL患者的疗效与安全性在一项Ⅱ期试验（NCT01421667）中被证实[16]。DLBCL患者的ORR为44％，CR率为17％，PR率为27％，中位PFS时间4个月，中位缓解持续时间5.6个月，CR患者的中位缓解持续时间16.6个月。中性粒细胞减少症和外周感觉神经病变是导致单药治疗方案剂量调整的主要AE。BV联合利妥昔单抗治疗组的ORR为46％，安全性和单药组类似。BV联合来那度胺治疗R/R DLBCL的有效性也被证实[17]，正在开展一项Ⅲ期ECHELON-3研究（NCT04404283），以评价BV联合来那度胺及利妥昔单抗治疗既往接受过2线及以上系统治疗的R/R DLBCL的疗效[18]。2022 ASCO大会上报道了Ⅲ期研究中安全性导入期的初步疗效数据，纳入10例患者，中位年龄70.5岁，6例患者之前接受过CAR-T细胞治疗，100％的患者出现≥1次治疗相关AE，最常见的是疲乏（50％）、贫血（40％）、便秘（40％）、食欲减退（30％）、腹泻（30％）等。8例患者由于AE导致药物剂量调整，原因包括贫血、中性粒细胞减少、外周神经病变和肺炎。患者ORR为70％，CR率为50％，中位缓解持续时间为5.6个月[19]。

4. Zilovertamab Vedotin（ZV）：ZV是由人源化IgG1单抗通过酶裂解连接子和抗微管细胞毒药物MMAE偶联而成的靶向ROR1 ADC药物。目前ZV单药治疗B-NHL患者的初步疗效还在研究中[20]。目前有限的数据显示，13例DLBCL患者的ORR为38.5％。15例既往接受了CAR-T/CAR-NK细胞治疗的患者仍有40％的肿瘤应答，提示ZV可作为细胞治疗失败之后的挽救治疗。最常见的安全性问题是仍然是血液学不良反应。2022年ASCO大会上公布了WAVELINE-003研究的设计，这是一项Ⅱ/Ⅲ期、开放标签、随机对照、剂量确定及扩展临床试验，研究中的队列A旨在评估ZV联合R-GemOx与R-GemOx治疗不能移植的R/R DLBCL患者的疗效与安全性[21]。队列A纳入既往接受过至少1次系统治疗失败、不适合接受ASCT或移植失败、CAR-T细胞治疗失败或不适合行CAR-T细胞治疗的患者。研究中的队列B旨在评估ZV联合BR与BR治疗不能移植的R/R DLBCL患者的疗效与安全性。队列B纳入之前接受过至少2次系统治疗失败、不适合接受移植或移植失败、CAR-T细胞治疗失败或不适合行CAR-T细胞治疗的患者。这项研究正在患者招募过程中。

二、单克隆抗体

治疗性单克隆抗体的出现为恶性B细胞淋巴瘤和实体瘤的治疗带来了革命性变化。继1997年美国FDA首次批准抗CD20单克隆抗体利妥昔单抗应用于B细胞淋巴瘤后，又陆续研发出奥法妥木单抗（Ofatumumab）和奥妥珠单抗（Obinutuzumab）。2020年靶向CD19抗原的tafasitamab获得FDA批准与来那度胺联合用于治疗R/R DLBCL。

Tafasitamab是靶向CD19的人源化Fc增强单克隆抗体，其Fc结构域进行了修饰，显著增强抗体依赖性细胞介导的细胞毒性和抗体依赖性细胞吞噬，从而改善肿瘤细胞杀伤的关键机制。

Tafasitamab单药治疗的ORR为26％，CR率仅为6％。常见不良反应包括血液学不良反应、肺炎、呼吸困难、疲乏和低钾血症等[22]。Tafasitamab与来那度胺联合方案（Tafa+Len）治疗R/R DLBCL患者，其ORR为60％，CR率为43％；中位随访33.9个月时，中位PFS时间为11.6个月，缓解具有持久性，中位缓解持续时间为43.9个月[23]–[24]。Tafasitamab在2020年获得美国FDA批准，联合来那度胺用于治疗不适合CAR-T细胞治疗的R/R DLBCL成人患者。两项真实世界研究RE-MIND[25]和RE-MIND2[26]研究证实Tafa+Len组合疗法的OS优于免疫化疗方案、来那度胺单药、Pola-BR、利妥昔单抗+来那度胺（R2）。目前也在探索tafasitamab联合苯达莫司汀与利妥昔单抗联合苯达莫司汀的疗效与安全性[27]。

三、双特异性抗体

双特异性抗体是指一个抗体分子与两个不同抗原或同一抗原的两个不同表位相结合的单克隆抗体。此独特作用机制可实现同时结合肿瘤细胞受体并招募细胞毒性免疫细胞，从而靶向杀灭肿瘤细胞。几乎所有的成熟T细胞表面都表达CD3分子，而B细胞肿瘤蛋白CD19和CD20作为B-NHL最成功的治疗靶点，对B系肿瘤具有良好的特异性，因此CD19×CD3和CD20×CD3成为了最受追捧的B-NHL治疗双抗靶点组合。

Blinatumomab是一种CD19×CD3双特异性抗体，双向结合B细胞表面CD19抗原与T细胞的CD3受体，通过激活内源性T细胞，导致CD19阳性的B细胞来源的肿瘤细胞定向裂解。目前blinatumomab在中国已经获批用于治疗成人及儿童复发或难治性前体B细胞急性淋巴细胞白血病。

Blinatumomab在DLBCL中的疗效也进行了很多探索。一项剂量递增Ⅰ期研究（NCT00274742）发现blinatumomab在复发难治B-NHL患者中的ORR为55％，CR率为36％[28]，主要的毒性反应为神经毒性。Blinatumomab在R/R DLBCL中的疗效通过一项多中心Ⅱ期试验（NCT01741792）得到验证[29]。经过15个月的中位随访，ORR为43％，CR率为19％，中位缓解时间为11.6个月，中位PFS时间为3.7个月，中位OS时间为5个月。进而将其向二线治疗推动，一线标准治疗失败后的R/R DLBCL患者中ORR达37％，完全代谢缓解率（CMR）为22％，中位PFS时间为8.4个月，56％的患者发生神经毒性[30]。

1. Mosunetuzumab（Mosun）：Mosun是一种靶向CD20×CD3、1∶1结构、IgG1样全人源化双特异性抗体。早期研究中Mosun单药治疗复发难治性B细胞NHL显示出有前景的疗效和安全性数据[31]。

Mosun单药治疗在R/R B-NHL患者中具有可控的安全性，并显示出持久性CR疗效，Ⅱ期研究的推荐剂量为1/2/60/60/30 mg[32]。另外，Mosun联合Pola治疗R/R B-NHL的疗效和安全性也在探索中。截至2021年3月15日，Mosun-Pola方案在DLBCL中ORR为73％，CR率为52％。整体人群中位缓解持续时间未达到。整体安全性可控，常见的超过3级治疗期间AE（TEAE）包括中性粒细胞减少和疲乏。细胞因子释放综合征（CRS）的发生率为18％，均为1～2级，在数据截止前均得到控制。

R/R侵袭性B细胞淋巴瘤中正在研究的双抗包括Glofitamab、Mosunetuzumab、Epcoritamab和Odronextamab等。Glofitamab是一种IgG1样全人源化、Fc段沉默处理、具有独特2∶1结构的CD20/CD3双特异性抗体，可同时与B细胞的CD20和T细胞的CD3结合，诱导T细胞的激活和针对肿瘤细胞的免疫反应，实现抗肿瘤效应。与Blinatumomab相比，Glofitamab具有更长的半衰期，更符合临床给药需求，既能提升缓解率，也具有良好的安全性。

一项正在进行的一项Ⅰ/Ⅱ期剂量递增研究（NCT03075696）评估了奥妥珠单抗预处理联合Glofitamab治疗CD20阳性的R/R DLBCL成人患者的疗效与安全性[33]–[34]。入组患者154例，其中大部分为多线治疗后难治性患者，中位治疗线数为3，59.7％的患者既往治疗≥3线。既往接受过CAR-T细胞治疗的患者占33.1％。中位随访12.6个月，研究达到主要终点，ORR为51.6％，CR率为39.4％。亚组分析中，既往治疗≥3线的患者CR率达44％，既往接受过CAR-T细胞治疗的患者CR率达35％，中位PFS时间4.9个月，中位OS时间11.5个月。最常见的AE是CRS。另外5.3％患者发生了神经毒性。Glofitamab治疗相关3～4级AE发生率41.6％，无治疗相关5级AE发生。最常见的AE是CRS（63.0％），3～4级CRS发生率为3.9％，38.3％患者发生神经毒性，3～4级神经毒性发生率为3.2％。

Glofitamab联合Pola方案展现了较高的缓解率，ORR为79.6％，CR率为51％[35]。在转化型滤泡性淋巴瘤、HGBCL中也观察到较多例患者缓解。虽然随访时间有限，中位随访时间仅3.7个月，25例患者中23例至数据截止时仍持续CR。支持进一步开展验证性研究。

目前Glofitamab在中国开展两项临床试验，一项是Ⅰ期评估既往接受两线或以上系统治疗失败的R/R DLBCL患者接受单次奥妥珠单抗预处理后接受Glofitamab单药治疗的药代动力学、安全性、耐受性和有效性。另一项是全球Ⅲ期研究评估Glofitamab联合吉西他滨+奥沙利铂和R-GemOx在既往接受≥1线系统治疗的R/R DLBCL患者中的安全性和有效性。

2. Epcoritamab：epcoritamab是一种在研的IgG1双特异性抗体，使用Genmab专有的DuoBody技术，epcoritamab同时结合T细胞上的CD3和B细胞上的CD20，并诱导T细胞介导的淋巴瘤B细胞杀伤作用。

EPCORE NHL-1研究是一项Ⅰ/Ⅱ期首个人体（first-in-human，FIH）剂量递增和队列扩展临床试验（NCT03625037）[36]，初步评估了epcoritamab治疗的安全性和抗肿瘤活性。2022年EHA大会上公布了Ⅱ期DLBCL扩展队列的数据。共纳入157例R/R DLBCL患者，患者既往中位治疗线数为3线。既往接受过CAR-T细胞治疗的患者占38.9％。中位随访10.7个月，ORR为63％，CR率为39％。CAR-T细胞治疗失败患者的ORR和CR率分别为54％和34％。常见的治疗相关AE为CRS（49.7％，≥3级2.5％）、发热（23.6％）、疲乏（22.9％）、中性粒细胞减少（21.7％）和腹泻（20.4％）。6.4％患者发生神经毒性，其中1例患者由于神经毒性而死亡。

继而，研究者开展了一项Ⅰ/Ⅱ期多队列临床研究EPCORE NHL-2（NCT04663347）评估epcoritamab联合其他方案治疗R/R DLBCL患者的疗效与安全性[37]。其中队列4研究旨在评估epcoritamab联合R-DHAX/C方案治疗可接受移植的R/R DLBCL患者的初步疗效与安全性。该研究共纳入29例患者，中位年龄58岁，72％的患者为一线治疗失败，66％患者为原发耐药，3例患者既往接受过CAR-T细胞治疗。在可评估疗效的26例患者中，epcoritamab+R-DHAX/C联合方案的CMR和部分代谢缓解率（PMR）分别为65％（17/26）和23％（6/26），中位随访5.8个月后，14例（54％）患者接受了ASCT，12例患者推迟或取消了ASCT计划。11例患者发生CRS（均为1～2级），1例患者发生2级神经毒性。

EPCORE NHL-2研究的队列5研究评估epcoritamab联合GemOx方案治疗不适合移植的R/R DLBCL患者的疗效与安全性[38]。共纳入26例患者，中位年龄71岁，62％的患者既往接受过≥2次系统性治疗，62％的患者为原发耐药，3例患者既往接受过CAR-T细胞治疗。在疗效可评估的25例患者中，epcoritamab+GemOx联合方案的CMR和PMR分别为60％（15/25）和32％（8/25），中位随访9.2个月后，15例（58％）患者停止了治疗，原因包括疾病进展（6例）、AE（5例）、死亡（3例）和退组（1例）。18例（69％）患者发生CRS（1例为3级，其余均为1～2级），1例患者发生3级神经毒性。62％的患者发生感染（3级以上占31％）。7例（27％）患者发生5级AE。

3. Odronextamab：Odronextamab是基于Veloci-Bi双特异性抗体平台生成的一款铰链稳定的、全人源化IgG4型CD20×CD3双特异性抗体。这一技术平台能够生成与天然抗体类似的全长双特异性抗体，具有与天然抗体类似的药代动力学特性及更低的免疫原性。此外，Odronextamab是IgG4型抗体，其Fc段功能微弱，可有效减少ADCC效应，避免T细胞损耗，从而发挥更强效持久的抗肿瘤作用。

Odronextamab单药治疗复发难治B-NHL患者的安全性和疗效已经得到初步验证[39]。DLBCL患者的推荐剂量为160 mg。最常见的3级或更严重的AE是血液学AE。在R/R DLBCL患者中，≥80 mg剂量组未接受过CAR-T细胞治疗的DLBCL患者（15例）的ORR和CR率为53％，中位缓解持续时间仍未达到，中位PFS时间为11.5个月。接受过CAR-T细胞治疗的DLBCL患者（30例），ORR为33.3％，CR率为27％，中位缓解持续时间仍未达到，中位PFS时间为2.9个月。

四、小结

随着ADC药物、单克隆抗体、双特异性抗体等抗体类新药的不断涌现，R/R DLBCL患者的治疗选择愈加丰富。ADC药物已被证明是一类非常有效的治疗手段，Lonca是迄今唯一在R/R DLBCL领域获批的ADC单药治疗方案，靶向CD19的单克隆抗体tafasitamab通过联合来那度胺顺利获批用于治疗R/R DLBCL患者。现阶段双特异性抗体已经在R/R DLBCL后线治疗中显示出较为不错的疗效与可耐受的安全性。我们也应关注到双特异性抗体临床研究的随访时间仍比较短，期待后续长期随访的数据更新，以进一步明确其长期生存获益与安全性。研究者已经开始基于合理的机制探索潜在的联合疗法，比如ADC药物与双特异性抗体的联合方案等。同时，由于R/R DLBCL获批新药的增加，如何确定不同药物的最佳应用顺序和组合将是下一步的研究方向。
